# The authors respond to feedback on *Cancer Cell-Memory Macrophage Hybrid Theory* for metastatic cancer cells

**DOI:** 10.3389/fonc.2026.1780597

**Published:** 2026-03-18

**Authors:** Jiaxi Wu, Chuo Jiang

**Affiliations:** 1 Retired, Shanghai, China; 2 Central Laboratories, Shanghai Clinical Research Center Xuhui Central Hospital, Chinese Academy of Sciences, Shanghai, China; 3 Office of Industrial Cooperation, Shanghai Institute of Nutrition and Health, Chinese Academy of Sciences, Shanghai, China; 4 School of Life Sciences, Shanghai University, Shanghai, China

**Keywords:** Coley’s toxin, human papilloma virus vaccine/HPV vaccine, immune checkpoint inhibitor, intratumoral bacterium/intratumoral microbe/intratumoral microbiome/intratumoral microbiota, metastasis, oncolytic virus/oncolytic bacterium, trained macrophage/memory macrophage, tumor hybrid cell

## Abstract

We have recently hypothesized that the hematogenous metastatic cancer cell of solid tumors is a hybrid between a primary cancer cell and a memory/trained macrophage (doi: 10.3389/fonc.2024.1412296). The hybrid cell respectively acquires mutator phenotype and overgrowth/hyperplasia property from the primary cancer cell and migratability/metastability from the memory/trained macrophage. We name this hypothesis *Cancer Cell-Memory Macrophage Hybrid Theory*. Since the publication of the article, a number of questions related to this *Theory* have been raised by colleagues in the oncology community, including intratumoral microbes and microbiomes/microbiotas, oncolytic viruses and bacteria, human papilloma virus vaccines, anti-cancer effects of γδ T-cells, and immune checkpoint inhibitors. The current article is prepared to address these issues. Additional to resolving questions like “Why metastatic cancer cells enter dormancy and can recur via stem-like self-renewal?”, the *Cancer Cell-Memory Macrophage Hybrid Theory* distinguishes itself from other carcinogenesis and metastasis hypotheses/theories by offering answers to many puzzling clinical features including metastasis of seemingly malignant parasitic cells within the human body, intracellular microbes (including viruses, bacteria, fungi, and parasites) within cancer cells, paradoxal effects (recurrence vs. regression) of microbes on cancer, contradictory immune effects of human papilloma virus vaccines between young and adult/senior females, and immune context-dependent effects (stimulatory and inhibitory) of T-lymphocytes on cancer cells. The *Theory* also predicts that quantitatively and functionally dampening innate macrophages that have hybridized with cancer cells (i.e., cancer cell-memory macrophage hybrids), should be explored as a fundamental anti-cancer strategy. The *Theory* further forecasts how to prepare an organotropic/tumoritropic Coley’s toxin-like anti-cancer microbe, which could potentially circumvent direct injection of microbial preparations into a tumor. A testable experiment that uses zebrafish larva models can potentially either validate or falsify the *Theory*.

## 1 Introduction

We have proposed that the metastatic cancer cell (metCaC) (**Box 1**) is a hybrid cell between a primary cancer cell (priCaC) and a memory/trained macrophage (memMφ) (**Box 1**), not other subtypes of macrophages (Mφs) (1). We refer this hybrid as a memory macrophage-like tumor hybrid cell (memφTHC) (**Box 1**), which is equivalent to metCaC (1). (These two terms are used interchangeably throughout this article.) This *Cancer Cell-Memory Macrophage Hybrid Theory* (or *Memory Macrophage Theory* hereafter) for hematogenous metastasis of solid tumors is founded on German pathologist Otto Aichel’s *Fusion Theory* for metCaCs, as well as biochemical, biological, and immunological resemblances between metCaCs/memφTHCs and memMφs. **Table 1** shows some of these resemblances, additional to others discussed throughout this article. Pawelek, Pawelek and Chakraborty, and Seyfried and Huysentruyt have reviewed that cancer cells (CaCs) express biochemical and biological traits of myeloid cells/macrophages (4, 104, 105). Actually, studies on a spontaneous metastasis animal model have also shown that “… detectible tumor cells at both the primary and distant metastatic sites were of fusion origin” (106). Moreover, “The macrophage origin of metastasis can account for most observations related to the disease” (4). As a matter of facts, tumor hybrid cells (THCs) hybridized with Mφs, have been explicitly studied by Aguirre et al. and Chou et al. (107, 108).

Box 1 **Glossary and abbreviations**

• APC: antigen-presentation cell.

• BCG: Bacillus Calmette-Guerin.

• CaC: cancer cell/cancerous cell. This term refers to any cancer cell/cancerous cell such as a primary cancer cell (priCaC), macrophage-like tumor hybrid cell (φTHC), and memory macrophage-like tumor hybrid cell (memφTHC)/metastatic cancer cell (metCaC).

• CTC: circulating tumor cell.

• DAMP: damage-associated molecular pattern.

• DED: dormant memMφ-effector Mφ-nest-generation dormant memMφ. Please refer to Section 8 of this article for details.

• DTC: disseminated tumor cell.

• EMT: epithelial-mesenchymal transition.

• GFP: green fluorescent protein

• ICD: immunogenic cell death.

• IFN: interferon.

• ILC: innate lymphoid cell.

• LPS: lipopolysaccharide.

• MAMP: pathogen-/microbe-associated molecular pattern.

• Mφ: macrophage.

• φTHC: macrophage-tumor hybrid cell/macrophage-like tumor hybrid cell/macrophage-cancer hybrid cell. This term refers to a cancer cell (CaC) that is hybridized with a macrophage (Mφ).

• metCaC: metastatic cancer cell. This term (i.e., metCaC) is equivalent to memory macrophage-like tumor hybrid cell (memφTHC) throughout this article.

• memMφ: memory/trained macrophage.

• memφTHC: memory macrophage-tumor hybrid cell/memory macrophage-like tumor hybrid cell. This term refers to a cancer cell (CaC) that is hybridized with a memory macrophage (memMφ). This term (i.e., memφTHC) is equivalent to metastatic cancer cell (metCaC) throughout this article.

• MHC: major histocompatibility complex.

• MSC: mesenchymal stem cell.

• NSCLC: non-small cell lung carcinoma.

• priCaC: primary cancer cell. This term refers to a cancer cell (CaC) that is NOT hybridized with macrophages (Mφ), or a macrophage-cancer cell hybrid cell (φTHC) that does NOT acquire or express migratory or metastatic properties from the macrophage (Mφ).

• RFP: red fluorescent protein

• PRR: patter recognition receptor.

• SNP: single nucleotide polymorphism.

• TAM: tumor-associated macrophage.

• TCR: T-cell receptor.

• THC: tumor hybrid cell. This term refers to a cancer cell (CaC) that is hybridized with other cells such as macrophages (Mφs), mesenchymal stem cells (MSCs), fibroblasts, and even other cancer cells (CaCs).

• TLR: Toll-like receptor.

• TME: tumor microenvironment.

**Table 1:** Shared properties by Mφs/memMφs/Mφ precursors and φTHCs/metCaCs/memφTHCs.

Shared properties between macrophages/memory macrophages and macrophage-like THCs/memory macrophage-likeTHCs/metCaCs	Mφs/memMφs/Mφ precursors: PubMed identification number (reference number)	φTHCs/metCaCs/memφTHCs: PubMed identification number (reference number)
Epithelial-mesenchymal transition (EMT)	31167103 (2), 33276594 (3)	23237552 (4), 37138326 (5)
Chemokines, chemokine receptors, and chemotaxis	15530839 (6), 30245686 (7)	21866172 (8)
Local invasion	10605792 (9), 28845386 (10)	21605699 (11)
Egressing or intravasation	17197241 (12), 28025672 (13)	17197241 (12), 28025672 (13)
Immune evasion	25457002 (14), 36010357 (15), 20427170 (16)	30963235 (17), 27644321 (18), 27866156 (19)
Extravasation or homing	34345249 (20), 25990461 (21)	17906932 (22)
Homing, organotropism pre-niche/niche, lodging, and colonization	38681642 (23), 22202039 (24), 25715759 (25), 34685700 (26), 36077265 (27), 39417006 (28)	22202039 (24), 25715759 (25), 31063756 (29), 22044002 (30), 30755309 (31)
Perivascular niches/regions associated with sinusoidal blood vessels or venules	30127389 (32)	30127389 (32)
Dormancy/quiescence or immunological dormancy	34146467 (33), 1733207 (34)	25118602 (35)
Awakening by microbes (via MAMPs) and abnormal autologous, allogeneic or heterologous cells (via DAMPs)	29287986 (36), 31749793 (37), 31998254 (38), 35911757 (39)	25118602 (35)
Stem cell-like features such as self-renewal	25457002 (14), 22900188 (40), 34911937 (41)	34911937 (41), 40371432 (42)
Hypoxia at the niche	21537168 (43), 28540498 (44)	19249648 (45), 22921864 (46)
Innervation	21537168 (43), 32508835 (47), 31017803 (48), 27177311 (49)	41009819 (50), 39831934 (51), 39825376 (52), 39934425 (53), 40376000 (54), 33334813 (55)
Macrophage surface biomarkers, including pattern recognition receptors (PRRs); and dual-labeled cancer cells (CaCs), including engulfed microbes, at primary site and distant sites	40529613 (56), 39516356 (57), 39035733 (1) (memMφ biomarkers)	37996690 (58), 39035733 (1) (memMφ biomarkers)
Signaling pathways, such as WNT and NOTCH	40529613 (56), 37706196 (59)	24439813 (60), 34482364 (61), 40422220 (62)
Purinergic signaling	28824882 (63), 26973651 (64), 33664731 (65), 34064383 (66)	32635260 (67), 37966629 (68)
Metabolism (such as glycolysis) and related epigenetic profile	23122286 (69), 28600802 (70), 36822175 (71), 40185732 (histone lactylation) (72), 40318634 (histone lactylation) (73), 26330802 (polyamines) (74)	23122286 (69), 29946772 (75), 40880049 (76), 36613471 (histone lactylation) (77), 34778253 (histone lactylation) (78), 40573249 (79) (histone lactylation), 35477776 (polyamines) (80)
N^6^-methyladenosime RNA methylation	34515345 (81)	37582735 (82)
Phagocytosis ability	23237552 (4)	23237552 (4)
Fusion ability	23237552 (4)	23237552 (4)
Autophagy	39439196 (83), 34467634 (84), 31892110 (85), 29163544 (86)	38390576 (87), 38494666 (88), 38253423 (89), 38902544 (90)
Intracellular microbes	32467386 (91), 35314111 (92), 37813921 (93), 38443279 (94), 19369951 (95)	32467386 (91), 36310856 (96), 40071093 (97), 39360320 (98)
Genetic/genomic alterations	32324846 (99), 12874786 (100)	34572863 (101), 29342134 (102), 20033424 (103)

**Figure 1:**
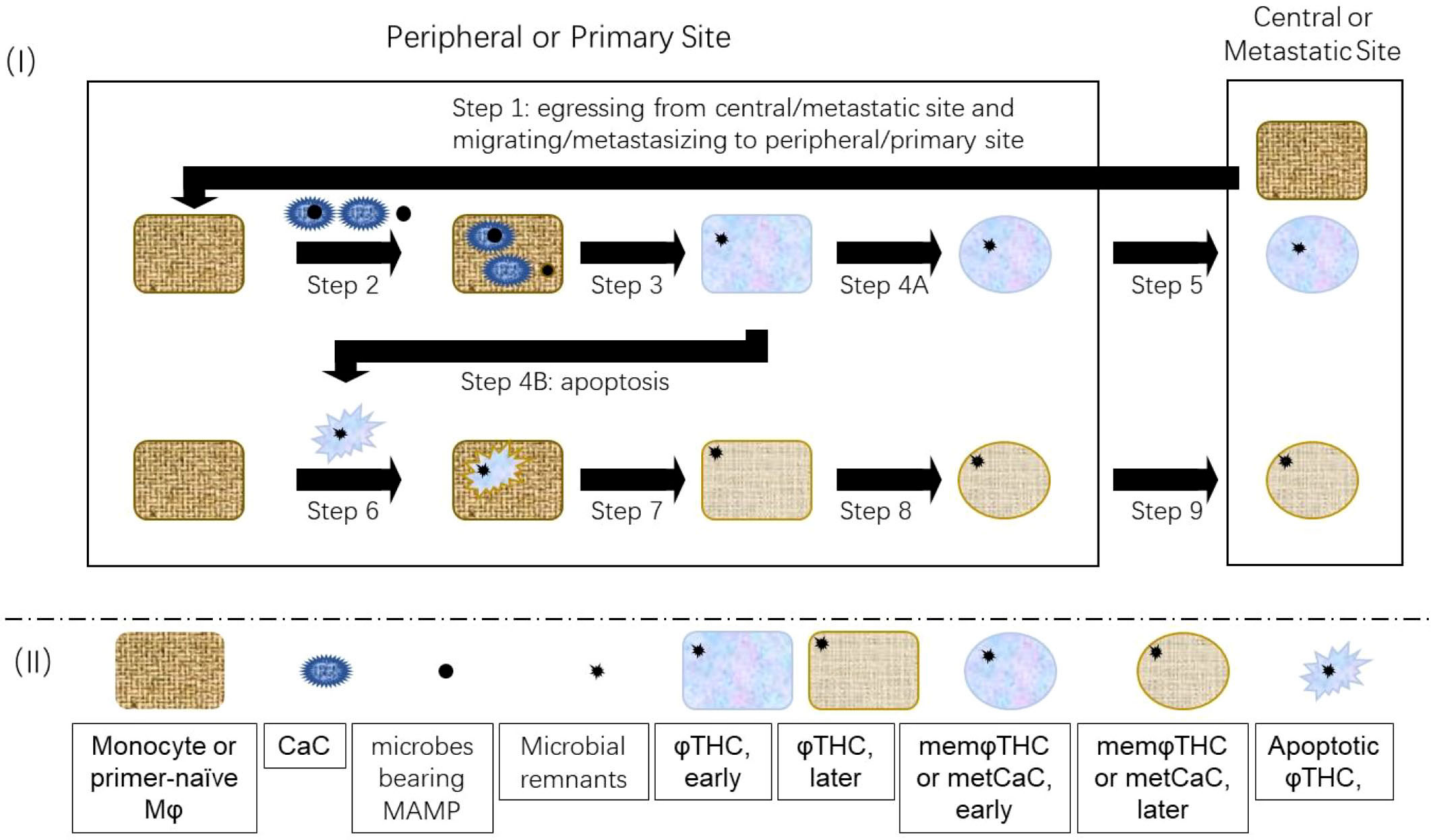
Model for the generation of heterogenous φTHCs/metCaCs/memφTHCs. As φTHCs/metCaCs/memφTHCs behave like conventional Mφs/memMφs, the entire metastatic process is similar to the migratory process of Mφs/memMφs. Step 1: Central monocytes or primer-/MAMP-naïve Mφs are activated by senescent/apoptotic priCaCs and migrate/metastasize to the primary site. Step 2: Senescent/apoptotic priCaCs with/without intracellular microbes prime/activate the monocytes and primer-/MAMP-naïve Mφs and then, are phagocyted by the monocytes and primer-/MAMP-naïve Mφs. Step 3: The monocytes and Mφs digest and degrade the engulfed priCaCs and intracellular microbes. Degradation-resistant priCaC constituents and microbial residues, such as DNA, persist within the Mφs, generating Mφ-priCaC or Mφ-priCaC-microbe hybrid cells (i.e., φTHCs). Step 4B: Some φTHCs enter apoptosis caused by inflammatory free radicals. Step 6: The apoptotic φTHCs are phagocytosed by Mφs. Step 7: The Mφs digest and degrade the engulfed φTHCs. Degradation-resistant remnants persist within the Mφs, generating heterogenous φTHCs. Steps 4A and 8: A limited number of φTHCs differentiate into memφTHCs (i.e., metCaCs) and some of these memφTHCs/metCaCs stay at the primary site as dormant tissue-resident memφTHCs. Steps 5 and 9: The other memφTHCs/metCaCs metastasize to distant organs, such as the bone, as dormant central/distant memφTHCs.

The *Memory Macrophage Theory* for hematogenous metastasis of solid tumors is briefly described as follows (**Figure 1**). Immunogenic cell death (ICD) of CaCs (109) and associated microbes elicit a non-sterile and/or “sterile” inflammation. As a part of the inflammatory response, Mφs either phagocytose or fuse with apoptotic or senescent CaCs, generating CaC-Mφ hybrid cells (φTHCs) (**Box 1**). Just like conventional Mφs and memMφs (110), a limited number of these φTHCs differentiate into memφTHCs (i.e., metCaCs) at the late inflammatory stage. The φTHCs consequently acquire mutator phenotype and overgrowth/hyperplasia property from the priCaCs and migratability/metastability from the memMφs (106, 111). Additionally, due to congenital DNA repair defects (Section 4.1 below), DNA repair machineries from both parental cells cannot process excessive/tetraploid DNA correctly, causing DNA and chromosome structural alterations (such as oncogene-associated chromosome translocations) to the hybrids, which drive the development of cancer (112, 113) and metastasis (1, 102, 114).

Therefore, our *Memory Macrophage Theory* further Dr. Otto Aichel’s *Fusion Theory* in several aspects. [I] Whereas cell fusion and phagocytosis are not identical cellular processes, we suggest that the hybrid can be generated not only through cell-to-cell fusion but also via phagocytosis of the priCaC or its debris (such as extracellular vesicles) by the Mφ/memMφ (4, 115), as well as via other ways of lateral/horizontal gene transfer (such as direct cell-to-cell contact) (116–119). As a matter of fact, Dr. Otto Aichel also considered that phagocytosis contributed to the generation of the fusion cell (104). [II] We specify that the “leukocyte” or “macrophage” referred by Dr. Otto Aichel and other scientists, is the memMφ, not other subtypes of Mφs, because it is the memMφ that can enter dormancy, be awakened from dormancy by primers (such as MAMPs and DAMPs), and self-renew like a stem cell. [III] We consider that DNA repair defects of the φTHC/memφTHC are the fundamental driver for the development (or multi-step carcinogenesis) of cancer (Sections 4.1 and 8.1 below).

The current article has been prepared primarily to address feedback regarding the *Memory Macrophage Theory* from scientist and clinician colleagues since the publication of the *Theory* (1). Additionally, several issues that are potentially important for BENCH RESEARCH/LABORATORY EXPERIMENTS on metCaCs/memφTHCs, have also been discussed (Section 22 below).

A virus, by definition, is not considered as a cell. A parasite is often not regarded as a microbe. However, in the current article, for convenience, “virus/viral” is sometimes also referred as “cell/cellular”, and “microbe” also includes “parasite”. For the same reason, the term “species” or “type” also encompasses genus, family, etc. of a microbe.

**Box 1** lists the abbreviations, as well as a number of terms and their connotations conferred specifically by this article.

The following Sections are organized to address feedback questions, on-by-one, on the *Memory Macrophage Theory*. As a result, it is not coherent between Sections. The current article is a supplement to the previous *Memory Macrophage Theory* article (1) and therefore, should probably be reviewed along with the previous *Theory* article.

## 2 Feedback question: what are shared properties between Mφs/memMφs and metCaCs/memφTHCs?

**Table 1** lists some of the shared biochemical, biological, and immunological properties between the Mφ/memMφ and the φTHC/memφTHC/metCaC. Cozzo et al. have reviewed that many of these properties result from the hybridization of CaCs with Mφs (120).

The metastatic cascade (including epithelial-mesenchymal transition (EMT), chemotaxis, local invasion, intravasation, immune evasion, circulating, extravasation, lodging/colonizing, dormancy, and stem cell-like self-renewal) as a result of the hybridization between CaCs and Mφs, has briefly been discussed earlier in the *Memory Macrophage Theory* article (1). The following sub-sections discuss some of these metastatic steps that have not been elaborated in the *Memory Macrophage Theory* article.

### 2.1 Pre-metastatic niches and metastatic niches

Before a CaC is metastasized to a specific distant site, a so-called pre-metastatic niche is formed (120), which can lodge the metCaC/memφTHC in the future. However, forming pre-niches/niches is not unique for metCaCs/memφTHCs. Rather, it is a common practice for leukocytes and their precursors (14, 121, 122), including Mφs (26, 27, 123, 124). As the metCaC/memφTHC is essentially a CaC that is hybridized with macrophages and acquires properties from the macrophages, it is not surprising that the metCaC/memφTHC also adopts the same pre-niche strategy for its future lodging at the distant metastatic site. As a matter of fact, the niche for Mφs and the niche for metCaCs/memφTHCs share similar structures (14, 24, 26, 27, 121, 122). Studies have also found that “tissue-resident Mφs provide a pro-tumorigenic niche to early human non-small cell lung carcinoma (NSCLC) cells” (125).

### 2.2 Epithelial-mesenchymal transition

Epithelial-mesenchymal transition (EMT) is considered as an initial and critical step for metastasis of epithelial cells. On the other hand, Mφs are categorized with mesenchymal cells. Epithelial cells that hybridize with Mφs immediately acquire mesenchymal features (4, 5, 104, 126, 127) as well as migratory/metastatic properties (125, 126). Actually, genes that are considered to be associated with EMT are often expressed not only by Mφs (4) but also by epithelium-Mφ hybrids (126).

### 2.3 Identical chemotactic laws for migration of Mφs and metastasis of metCaCs

Migration of conventional Mφs follows fundamental physical and chemical laws, i.e., migrating cells must express chemokine receptors and must migrate along the concentration gradient of chemokines (128). As a hybrid between a CaC and a Mφ, the metCaC/memφTHC follows the same laws (129). Among many cytokines and chemokines (**Table 1**), memMφ biomarkers, such as CD204, CD206, CD163, and PRRs (1), indicate that metCaCs/memφTHCs expressing these biomarkers can enter dormancy to fulfill the metastatic process/cascade.

### 2.4 Immune evasion

According to the *Memory Macrophage Theory*, metCaCs/memφTHCs are essentially Mφs that have hybridized with CaCs and therefore, are recognized by immune cells as “one of our own” (126). As a result, like conventional Mφs (15, 16), metCaCs/memφTHCs express both immune checkpoint ligands and receptors (17), likely to respectively avoid being mistakenly recognized as non-self-cells and cleared by other autologous immune cells (58) and to avoid erroneously phagocytosing other self-cells.

## 3 Feedback question: is tumor microenvironment required for the generation of metCaCs/memφTHCs?

A century ago, Dr. Stephen Paget proposed the *Seed and Soil Theory* which states that tumor microenvironment (TME) contributes to metastasis of CaCs (130). The *Memory Macrophage Theory* re-iterates the importance of the TME (i.e., “soil”) which induces the expression of the intrinsic property of the priCaC (i.e., “seed”). The hematogenous metCaC is a hybrid of the priCaC and the Mφ; the latter is an important player of the TME. As a matter of fact, it is the M2 tumor-associated macrophage (TAM) that are closely related to the generation of the memφTHC/metCaC (126, 127, 131, 132) in the reparative stage of an inflammation (133). Without the Mφ, the priCaC cannot acquire the migratability/metastability, such as dormancy and stem cell-like self-renewal at the distant metastatic site.

## 4 Feedback question: what is the evidence that cancer is a DNA repair-defective disease?

### 4.1 Cancer as a DNA repair-defective disease

MemMφs are universally present for all humans whereas cancer patients are much rarer. We attribute this discrepancy to DNA repair defects because cancer almost always occurs to individuals with congenital DNA repair defective syndromes (134–138) and single-nucleotide polymorphisms (SNPs) in DNA repair genes (139) whereas the vast majority of humans do not have these genetic variations. **Table 2** summarizes relationships between congenital DNA repair-defective syndromes and associated types of cancers. As a matter of fact, we have earlier proposed that cancer is a DNA repair-defective disease (140, 141) (**Box 2**).

**Table 2:** Cancer-prone syndromes, associated genes and types of cancers.

Syndromes/diseases	Mutated DNA repair gene	Involved DNA repair pathways	Cancer types	References
Ataxia-telangiectasia	*ATM*	DNA double-strand break repair	Epithelial solid tumor, lymphoma, T-cell leukemia	(134, 135)
Nijmegen breakage syndrome	*NBS1*	DNA double-strand break repair	Lymphoma	(134, 135)
Bloom syndrome	*BLM* (also known as *RECQL3*)	DNA double-strand break repair	Osteosarcoma; Wilm’s tumor; epithelial, hematopoietic, lymphoid, connective tissue, germ cell, nervous system, and kidney malignancies	(134, 135)
Werner syndrome	*WRN* (also known as *RECQL2*)	DNA double-strand break repair	Melanoma, soft-tissue sarcoma, thyroid cancer, meningioma, osteosarcoma	(134, 135)
Rothmund-Thomson syndrome	*RECQL4*	DNA double-strand break repair	Non-melanocytic skin tumor, osteosarcoma	(134, 135)
Fanconi anemia	*FANC-A* to *FANC-M*	DNA double-strand break repair	Squamous cell carcinoma; acute myelogenous leukemia; head and neck, gastrointestinal tract, and genital tract cancers	(134, 135)
Hereditary non-polyposis colorectal cancer/Lynch syndrome	*hMSH1*, *hMSH2*, *hMSH6*, *hMLH3*, *hPMS1*, *hPMS2*	Mismatch repair	Colon, endometrial, ovarian, stomach, kidney (transitional cell carcinoma), small intestinal, hepatobiliary tract, brain (glioblastoma), and skin cancers	(134, 135)
Muir-Torre syndrome	*hMSH1*, *hMSH2*	Mismatch repair	Sebaceous carcinoma; colon. endometrial, ovarian, stomach, kidney, and small intestinal cancers	(134, 135)
Turcot syndrome	*hMSH1, hPMS2*	Mismatch repair	Medulloblastoma; glioma; lymphoma; colon. endometrial, ovarian, stomach, kidney, and small intestinal cancers	(134, 135)
Xeroderma pigmentosum/DeSanctis Cacchione syndrome	*XPA* to *XPG*, *XPV*	Nucleotide excision repair (*XPA* to *XPG*), translation DNA synthesis (*XPV*)	Basal cell carcinoma, squamous cell carcinoma, melanoma, ocular cancer	(134, 135)
*MYH*-associated polyposis	*MUTYH*	Base excision repair	Colorectal cancer	(135)
Li-Fraumeni syndrome	*TP53, CHEK2*	DNA damage checkpoint	Soft-tissue sarcomas; breast cancers; leukemias; osteosarcoma; melanoma; colon, pancreas, adrenal cortex, and brain cancers	(135)
Hereditary breast-ovarian cancer syndrome	*BRCA1*, *BRCA2*		Breast, ovarian, and prostate cancers	(135)

Box 2 **Cancer as a DNA repair-defective disease**

The proposal is scientifically founded on three pieces of evidence: genetic, biochemical, and biological evidences (140, 141). The shared genetic feature of many congenital syndromes that predispose humans to cancer, is altered/mutated DNA repair genes (**Table 2**) (134–138). Biochemically, DNA repair proteins encoded by these mutated genes are defective and cannot restore damaged DNA to its original structures with high fidelity (134–136). Biologically, CaCs often show a mutator phenotype, such as single nucleotide alterations (polymorphisms and mutations) and chromosome aberrations (142), that is, CaCs cannot faithfully restore DNA structures, which is indictive of erroneous DNA repair/DNA mis-repair, or defective DNA repair. The original priCaC, prior to its hybridization with the Mφ, acquires mutator phenotype as a result of its genetically inherited defective DNA repair pathways and subsequent activation of alternative low-fidelity backup DNA repair pathways (140).

Both CaCs and Mφs genetically inherit DNA repair defects (**Table 2**), which are critical to the generation of φTHCs/memφTHCs/metCaCs during apoptosis. Apoptosis of CaCs leads to DNA fragmentation. The fragmented DNA can be re-ligated and consequently, the cells undergoing apoptosis can sometimes be rescued from cell death and revive (143, 144). In the case of DNA repair-defective cells, apoptosis-associated DNA fragments engulfed by Mφs are mis-relegated as a result of defective DNA repair (145, 146), causing a large scale of chromosome structural aberrations or so-called chromothripsis (116, 143, 147–149), including oncogene-associated chromosome translocations (143, 146, 150). The resulting genomic instability promotes metastasis of cancer (101–103). Indeed, the genome of CaCs is assembled from DNA fragments all at once in a limited step-wise number of events, i.e., multi-step carcinogenesis (Section 8.1 below), as in the case of chromothripsis (151).

Our view that cancer is a DNA repair-defective disease has recently been echoed by other research groups (138, 139, 152–154).

It should be reminded that occasional chromosome rearrangements, NOT chromothripsis, can also occur to cells that do NOT necessarily undergo apoptosis.

It should also be reminded that while genetically DNA repair-defective cells are prone to single nucleotide and chromosome structural alterations, normal DNA repair-proficient cells can also acquire these DNA structural alterations when the amount of DNA damage exceeds DNA repair capacity of a DNA repair-proficient cell (140), which is the likely to be the driving force for evolution.

It should further be reminded that a number of cancer-related microbes express microbial proteins that can interfere with host DNA repair (155–158).

### 4.2 Uncontrolled/unlimited proliferation as a compensatory mechanism of DNA repair-defective cells to address excessive DNA damage-induced cell death

One of the hall marks of cancer is the so-called “uncontrolled” or “unlimited” proliferation of CaCs. As a matter of fact, neoplasia/cancerization of parenchymal cells of an organ is almost always preceded by hyperplasia, metaplasia, and dysplasia. According to the *Alternative DNA Repair Theory* (140, 141) and the current *Memory Macrophage Theory* (1 and current article), the uncontrolled proliferation is a COMPENSATORY mechanism of parenchymal cells to address excessive cell death of an organ. Comparing to normal DNA repair-proficient cells, DNA repair-defective cells are much more susceptible to DNA damage. Exposure to intensive and/or persistent DNA damage (such as massive radiation and chronic inflammation), can cause death to a large number of DNA repair-defective parenchymal cells, leading to the compromise of the integrity (i.e., size/mass and subsequent functionality) of the organ. To catch up with the speed of the death of the cells, remaining alive DNA repair-defective parenchymal/stem cells compensatorily undergo hyperplasia, at the price of DNA fidelity, to maintain the integrity of the organ. That is to say, between the worse (cell transformation) and the worst (immediate compromised integrity of the organ that can lead to imminent life-threatening consequences) choices, the DNA repair-defective parenchymal/stem cells choose the former. Long-term exposure to DNA-damaging agents can ultimately reprogram genetic and epigenetic landscapes of the cells (159), conferring a phenotype of uncontrolled cell proliferation, even in the absence of DNA damage. Therefore, hyperplasia of parenchymal cells of an organ is generally considered as a pre-neoplastic/pre-malignant/pre-cancerous/pre-tumorigenetic lesion.

Cells of xeroderma pigmentosa, which are defective in nucleotide excision DNA repair, are sensitive to sunlight/UV-light and die quickly (160). To avoid exposure of under-skin tissues to external environmental infective microbes, xeroderma pigmentosum cells undergo hyperplasia, at the sacrifice of DNA fidelity (161, 162), and are eventually cancerized. Likewise, the development of many other types of cancers, such as gastric cancer (163, 164), colon cancer (165), pancreatic cancer (166), lung cancer (167, 168), liver cancer (169), biliary cancer (166, 170), ovarian cancer (171), breast cancer (172), head and neck cancer (173, 174), and others (159), is also initiated with hyperplasia of parenchymal cells under various DNA damaging conditions (such as chronic inflammation) before these cells evolve into neoplastic cells. Therefore, uncontrolled cell proliferation is a CONSEQUENCE of DNA repair defects, rather than an initial event for cancer development.

It should be remined again that single nucleotide polymorphisms/mutations or chromosome aberrations (including chromosome translocation-associated oncogenes), which mean loss of DNA fidelity, are indicative of erroneous repair/mis-repair of DNA, most likely to occur as a result of defective DNA repair (140, 141). (The proof-reading 3’-5’ exonuclease of the DNA polymerase is essentially a DNA repair activity, on-site during DNA replication).

### 4.3 Immunodeficiency of patients with DNA repair defects and anti-cancer immunotherapy

Many DNA repair proteins are involved in V(D)J recombination, which is required for the generation of T-cell receptors (TCRs) of T-lymphocytes and antibodies of B-lymphocytes/plasma cells (175, 176). Therefore, DNA repair-defective cancer patients often show an immunodeficient phenotype (1, 175, 176). Defective DNA repair could potentially have an impact on the efficacy of adaptive immune therapy against cancer, which relies on cytolytic or cytotoxic activities of T-/B-lymphocytes.

## 5 Feedback question: do most, if not all, CaCs express Mφ biomarkers?

It has been observed that, depending on detecting techniques and Mφ biomarkers that are selected for the analysis (Section 22.1 below), up to 100% of circulating tumor cells (CTCs), disseminated tumor cells (DTCs), and/or metCaCs at the primary and metastatic sites, are dual-positive for original priCaCs and Mφs (**Table 1**) (4, 5, 101, 107, 177–181). Therefore, it seems to be a general phenomenon that metCaCs are Mφ-like hybrids and express Mφ biomarkers, which is consistent with the *Memory Macrophage Theory*.

## 6 Feedback question: are both conventional Mφs and metCaCs hybrids that hybridize with engulfed microbes, autologous and non-autologous cells, and/or other substances?

### 6.1 Conventional Mφs as hybrids hybridized with engulfed microbes, autologous and non-autologous cells, and/or other substances

Conventional Mφs phagocytose microbes, exogenous substances, non-autologous cells (such as allogeneic transplanted cells), dying or senescent autologous cells, and cellular debris. Most, if not all, Mφs cannot thoroughly degrade engulfed materials (39) and, therefore, almost always contain constituents or debris of engulfed cells and substances (**Table 1** and references therein), including debris of CaCs (182, 183). Remnants of engulfed viruses, bacteria, fungi, and exogenous substances are easily identified within Mφs (94, 95, 184–188), including live microbes (95). Moreover, DNA of some engulfed microbes is also often found to be integrated into the genome of Mφs (189–192). As a hybrid cell is defined as a cell that possesses genetic/genomic DNA from more than one cells, many Mφs, by definition, are hybrid cells. Additional to being hybrid cells, Mφs may also hold non-DNA substance. Foam cells, which are often observed among atherosclerotic patients, are Mφs that contain un-degraded lipids. Therefore, ALL Mφs are potentially hybrid cells (DNA from two or more cells) or hybrids (exogenous non-DNA substances).

Additional to phagocytosis, Mφs can also acquire DNA of other cells (autologous, allogeneic, and heterologous) and microbial DNA via cell-to-cell fusion (116, 125, 144, 177) and other ways of lateral/horizontal gene transfer (such as direct cell-to-cell contact) (116–119). Moreover, hybridization of cells with Mφs can facilitate future hybrid-Mφ fusion, probably due to shared Mφ cellular components between the hybrids and the Mφs.

### 6.2 MetCaCs as CaC-Mφ hybrids hybridized with engulfed microbes, autologous and non-autologous cells, and/or other substances

Like conventional Mφs/memMφs, φTHCs/memφTHCs/metCaCs can also phagocytose (125) or fuse with other cells, including microbial cells (125). Indeed, intratumoral microbes or microbial constituents are often found to be present within tumor cells (97, 193–199). As a matter of fact, many so-called intratumoral/intratumor microbes are present inside tumor cells (93, 193, 194, 198) and some of these intracellular microbes are even alive (93, 193, 194, 198). Moreover, DNA of these intracellular microbes, including viral and bacterial DNA, can integrated into the genome of metCaCs (96, 191–193, 200–204). As discussed below (Sections 10 and 11 below), these intratumoral microbes, which are often tumor-specific, can be used for patient- and tumor-tailored anti-cancer microbial preparations.

### 6.3 Contribution of cell-to-cell fusion, phagocytosis, and other lateral/horizontal gene transfer to φTHCs/memφTHCs/metCaCs

Hybridization between two cells can be mediated via cell-to-cell fusion, phagocytosis, and other lateral/horizontal gene transfer (4, 115–119, 125, 144, 177). Readers who are interested may refer to the literature for a thorough discussion.

In the literature, whenever a CaC-Mφ hybrid cell/φTHC (which is often identified with dual-biomarkers for both CaCs and Mφs) is found, it is often termed as a “fused cell” or a “fusion cell”. Moreover, its occurrence is intuitively attributed to cell-to-cell fusion. However, rarely have any of these studies provided any DIRECT evidence to support that cell-to-cell fusion process does occur and contribute to the generation of the hybrid cell (125). Since the metCaC can phagocytose like the conventional Mφ, and the primary resort for the conventional Mφ to form a hybrid cell is by phagocytosis as discussed above (Section 6.1 above), it is likely that phagocytosis is an important strategy for the generation of the φTHC/memφTHC/metCaC, additional to cell-to-cell fusion. As a matter of fact, Dr. Otto Aichel also considered that phagocytosis contributed to the generation of fusion cells (104).

However, whether it is the fusion, phagocytosis, and/or other hybridizing process (116–119, 125) that contribute to the generation of the memMφ/memTHC/metCaC, remains to be investigated.

## 7 Feedback question: why cancer behaves like a wound that never heals and what is its relationship with DNA repair defects of CaCs?

Cancer is proposed to be a wound that never heals (205). As discussed above (Section 4.1 above), cancer is a DNA repair-defective disease. Therefore, comparing to normal DNA repair-proficient cells, CaCs are more liable to cell death and senescence under DNA-damaging conditions. The dead and senescent CaCs often elicit an inflammatory response (including activation of Mφs/memMφs and φTHCs/memφTHCs/metCaCs) that is overwhelmed with free radicals for clearing dead and senescent cells. The overwhelming free radicals further damage DNA of other live cells nearby, leading to their death and senescence, which subsequently provoke a second inflammation. Therefore, a vicious cycle, “cell death/senescence––––inflammatory free radicals––––DNA damage by free radicals to other live cells nearby––––death/senescence of other live cells nearby––––a second inflammation”, is generated. This vicious cycle does not seem to be easily terminated by itself. Moreover, episodes of these inflammations can overlap with one another, presenting cancer as a never-healing wound.

Contrasting to DNA repair-defective CaCs, normal DNA repair-proficient cells are more resistant to free radical-induced DNA damage and, normally, resolution of an inflammation ensues.

## 8 Feedback question: what are underlying mechanisms for multi-step carcinogenesis, field cancerization, and degree of differentiation of CaCs?

**Figure 2:**
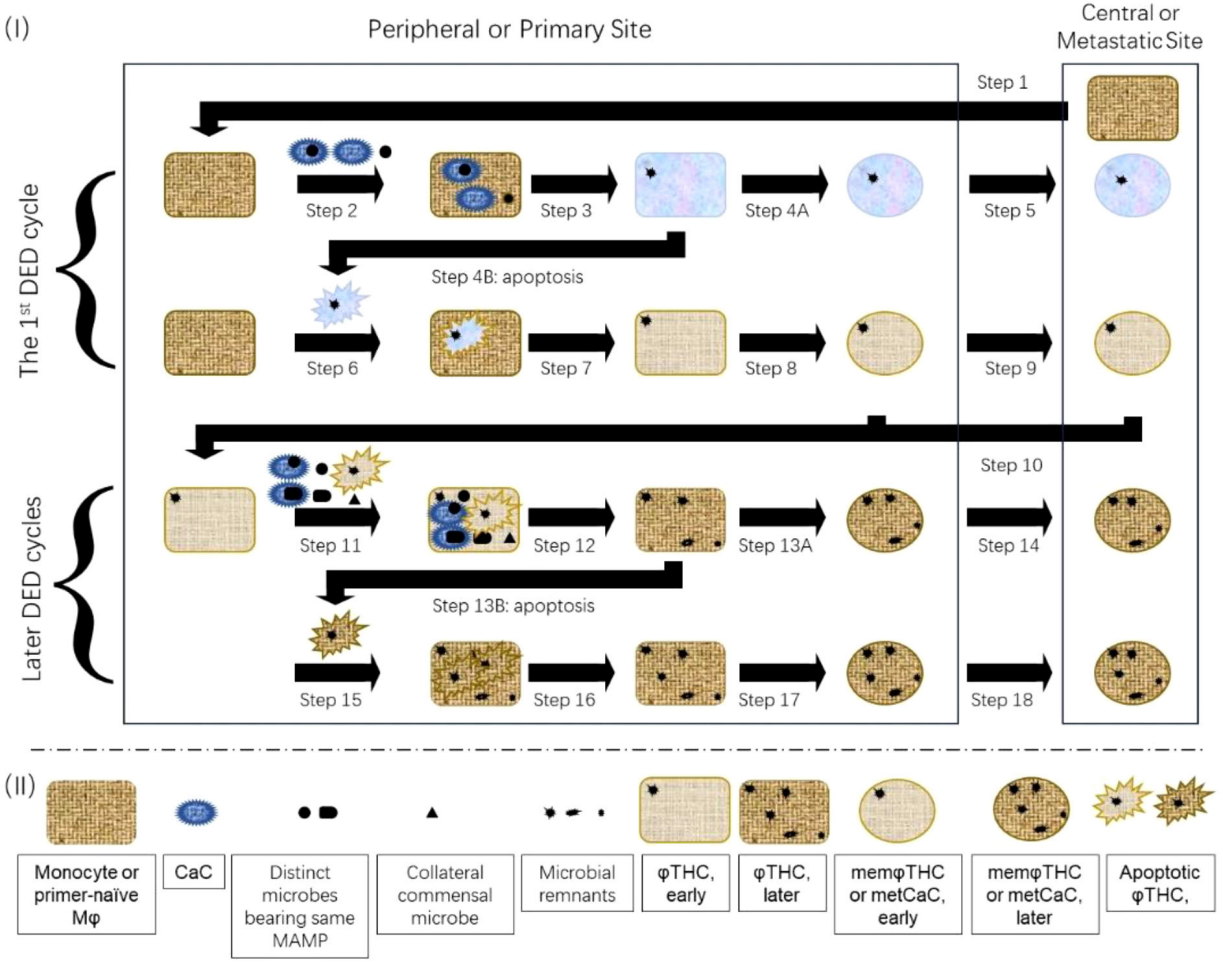
The “dormant memMφs/memφTHCs/metCaCs-effector Mφs/φTHCs-nest-generation dormant memMφs/memφTHCs/metCaCs (DED)” cycle. As φTHCs/metCaCs/memφTHCs behave like conventional Mφs/memMφs, the entire metastatic process is similar to the migratory process of Mφs/memMφs. Step 1 to Step 9:Step 1 to Step 9, which are descried in **Figure 1**, compose the first DED cycle. Step 10 to Step 18: Step 10 to Step 18 compose the second or later DED cycles. Step 10: Acquainted primers/MAMPs awaken dormant central and tissue-resident memφTHCs/metCaCs which subsequently differentiate into next-generation effector φTHCs. Step 11: The next-generation effector φTHCs phagocytose not only any cells/microbes that bear the same types of primers/MAMPs, but also other microbes, such as commensal microbes, in the vicinity. Step 12: The next-generation effector φTHCs digest and degrade the engulfed CaCs and microbes. Degradation-resistant CaC constituents and microbial residues, such as DNA, remain within the next-generation effector φTHCs, not only generating heterogenous effector φTHCs (comparing to the first-generation effector φTHCs) but also enriching the intratumoral microbiome. Step 13B: Some next-generation effector φTHCs enter apoptosis caused by inflammatory free radicals. Step 15: The apoptotic effector φTHCs are phagocytosed by other φTHCs (as well as Mφs). Step 16: The other φTHCs digest and degrade the engulfed apoptotic effector φTHCs. Degradation-resistant remnants persist within the next-generation φTHCs. Step 13B and Step 17: A limited number of the next-generation effector φTHCs differentiate into next-generation dormant memφTHCs/metCaCs as next-generation tissue-resident memφTHCs at the primary site. Step 14 and Step 18: The other next-generation memφTHCs/metCaCs metastasize to the distant metastatic site as next-generation central/distant memφTHCs. Please note that earlier-generation φTHCs are phenotypically and morphologically closer to priCaCs (as shown by a mixture of light blue and light purple colors) whereas later-generation φTHCs are closer to Mφs (as shown by light and dark brown color). The darker the brown color, the closer the φTHCs are to the Mφs.

Conventional dormant/quiescent memMφs can be awakened by acquainted MAMPs/DAMPs and differentiate into effector Mφs. At the end of an inflammation, a limited number of these effector Mφs re-differentiate into next-generation memMφs which subsequently enter dormancy/quiescence (1 and references therein). As a result, one round of the “dormant memMφs––––effector Mφs––––nest-generation dormant memMφs (DED)” cycle is fulfilled. However, the next-generation memMφs can be awakened again by acquainted MAMPs/DAMPs of another infection, initiating another round of the DED cycle (**Figure 2**) (1). Theoretically, this DED cycle can be repeated for numerous rounds.

Essentially as a Mφ that is hybridized with CaCs, the φTHC/memφTHC/metCaC inherits the property of conventional Mφs/memMφs and therefore, can also repeatedly undergo the same DED cycle (**Figure 2**), except that the effector φTHC/memφTHC/metCaC is DNA repair-defective and may mis-relegate apoptotic DNA fragments, causing chromothripsis (i.e., chromosome aberrations) (Section 4.1 above) during each DED cycle.

The DED cycle of the φTHC/memφTHC/metCaC may be related to several features of CaCs.

### 8.1 Multi-step carcinogenesis and field cancerization

For one DED cycle, the engulfment of an apoptotic CaC by a DNA repair-defective φTHC and the subsequent mis-religation of apoptotic DNA fragments can potentially lead to chromothripsis, causing chromosome structural alterations in the next-generation φTHC/memφTHC/metCaC (Section 4.1 above) (1). Therefore, each round of the DED cycle may introduce additional chromosome structural alterations (such as translocation of pro-oncogenes) into the genome of the φTHC/memφTHC/metCaC. This is in alignment with the *Multi-Step Carcinogenesis Theory* (206), that is, each DED cycle contributes to one step (i.e., an oncogenic DNA/chromosome alteration) of the multi-step carcinogenesis. Multiple rounds of the DED cycle can sequentially cause multiple genetic alterations to the φTHC/memφTHC/metCaC and consequently, multiple steps along the development of cancer. Together with MAMP-/PAMP- or PRR-specific clones of φTHCs/memφTHCs/metCaCs (1), a tumor may consist of multiple and heterogenous CaC clones that are PRR-specific and at distinctive steps of the multistep carcinogenesis, or so-called field cancerization (207, 208).

### 8.2 Degree of cancer cell differentiation

We have discussed that the respective contribution of either parental Mφ or parental priCaC to the final composition of the φTHC/memφTHC/metCaC varies from one hybrid to another (1), which may determine the degree of differentiation of the φTHC/memφTHC/metCaC.

As φTHCs/memφTHCs/metCaCs repeatedly undergo the DED cycle for multiple rounds, constituents of the original priCaC are diluted out whereas the relative proportion of Mφs is likely to be expanded (**Figure 2**). As a result, the resulting φTHCs/memφTHCs/metCaCs accrue more DNA and chromosome structural variations as a result of apoptotic-associated chromothripstic events (Section 8.1 above), behave less like the original priCaC but more like Mφs, and consequently, are more poorly differentiated (assuming other variables, such as types and subtypes of cancer, are the same). Clinical studies support this theory (209).

Rarely, when constituents of the phagocytosed microbe, so-called the intratumoral microbe (Section 10.1 below for discussion of intratumoral microbes) predominate (as a result of the resistance of the microbe to macrophagic degradation), the resulting microbe-containing φTHC/memφTHC/metCaC may morphologically present itself as a cell that resembles the microbe more than a human cell. As a result, a seemingly cancerous “parasite cell” metastasizing within the human body occurs (210).

A potentially relevant issue is the trans-differentiation of conventional Mφs (211–216) and φTHCs (217) into epithelioid cells and fibroblasts/myofibroblasts, which has been observed for decades. *Vice vasa*, some epithelial cells and fibroblasts possess phagocytosing ability (218). It remains to be investigated whether these so-call “epithelial cells”/”fibroblasts” are actually hybrids between Mφs/φTHCs and epithelial cells/fibroblasts, and consists of much more constituents of the epithelial cells/fibroblasts than those of the Mφs (4, 127, 146). In other words, these so-call “epithelial cells”/”fibroblasts” are actually epithelial cell-/fibroblast-Mφ hybrids but phenotypically and/or morphologically appear as epithelial cells/fibroblasts. Indeed, it has been suggested that, without detailed genetic analysis, a fusion cell might be interpreted as a de-differentiated or trans-differentiated cell (116).

It should be reminded that additional to Mφs, CaCs may also hybridize with other types of cells such as mesenchymal stem cells (MSCs) and fibroblasts (4, 219), generating other types of tumor hybrid cells (THCs), which may also contribute to the differentiation of CaCs.

A separate issue is that the Mφ can be used as a vehicle to disseminate latent microbes (such as *M. tuberculosis*, *F. nucleatum*, and human immunodeficiency virus/HIV) to distant organs/sites of the body (**Table 1**) (95, 220–224). As a matter of fact, this Mφ, by definition, is a hybrid cell between a Mφ and the microbes (Section 6.1 above).

## 9 Feedback question: how can *Memory Macrophage Theory* reconcile early metastasis with accrual of multiple mutations for the development of cancer?

Metastasis was originally thought to occur only after a sufficient number of genetic mutations were accrued, the so-called *Progression Model* (225). On the hand, it was later found that CaCs could metastasize at the early stage of carcinogenesis (225). The *Memory Macrophage Theory* seems able to reconcile the contradictory observations between the *Progression Model* and early metastasis as follows. As long as a priCaC hybridizes with the Mφ (i.e., φTHC), which can occur due to apoptosis of the priCaC induced by microbial infection or DNA damage at the early stage of cancer development, the φTHC acquires and expresses chemokine receptors or cellular components from the Mφ, and potentially has the migratory/metastatic ability. Therefore, CaCs can metastasize at the early stage of carcinogenesis. Moreover, as the φTHC undergoes rounds of the DED cycle (Section 8 above), it accrues more single nucleotide and chromosome structural alterations (Section 8.1 above) as well as more constituents from the Mφ to be more migratory/metastatic (Section 8.2 above).

## 10 Feedback question: how can *Memory Macrophage Theory* interpret organ-/tumor-specificity of intratumoral microbiome and integration of microbial DNA into host genome?

Various types of microbes, including viruses, bacteria, and/or fungi, as well as their remnants such as microbial DNA, are often found within a tumor (Section 6 above) (97, 193–199). Many intratumoral microbes are located inside CaCs of the tumor (93, 193, 194, 198) and some of these intracellular microbes are even alive (93, 193, 194, 198). Moreover, DNA of the intratumoral microbes, such as viral and bacterial DNA, has been found to be integrated into the genome of φTHCs/memφTHCs/metCaCs (96, 191–193, 200–204). These microbes are called intratumoral/intratumor microbes (such as intratumoral/intratumor bacteria) whereas entire types of microbes and their genetic materials within a tumor are called intratumoral/intratumor microbiota and microbiome, respectively (226).

It should be reminded that, as discussed above (**Table 1**), the conventional Mφ can also be used as a vehicle to disseminate latent microbes (such as *M. tuberculosis*, *F. nucleatum*, and human immunodeficiency virus/HIV) to distant organs/sites of the body (95, 220–224).

### 10.1 Complexity of intratumoral microbiome

The DED cycle (Section 8 above) may contribute to the complexity of the intratumoral microbiome (**Figure 2**) for the following reasons. [I] Owing to the specificity between the PRR and the MAMP, a memMφ/metCaC/memφTHC with a specific PRR (such as TLR4) can be activated by a specific MAMP (such as LPSs). On the other hand, many types/species of microbes (such as gram-negative bacilli) share a same type of MAMP (such as LPSs). Therefore, the same memMφ/metCaC/memφTHC can be activated and engulf these distinct microbes during different infectious/inflammatory episodes, leading to multiple types/species of microbes within the same memMφ/metCaC/memφTHC (**Figure 2**). [II] A memMφ/metCaC/memφTHC that is activated by a specific MAMP, can non-specifically engulf not only the pathogenic microbe that elicit an inflammation but also other bystander microbes on-site and in the vicinity of the inflammatory site as collaterals, irrespective of whether they are commensal or pathogenic or whether they share the same MAMP (**Figure 2**). Therefore, following multiple rounds of the DED cycle, a metCaC/memφTHC is enriched with these various types of pathogenic and commensal microbes.

### 10.2 Organ-/tumor-specificity of intratumoral microbiome

Additional to the complexity, the intratumoral microbiome is also patient- and tumor-specific (97, 194, 195, 227–230). It is known that commensal and pathogenic microbes at distinct anatomic sites (e.g. bowels vs. skins) of the body and even distinct segments/areas within the same organ (e.g. ascending colon vs. sigmoid colon), can be distinct. Tumors developed at a specific organ often co-live with these organ-specific microbes. Like Mφs, φTHCs can engulf and harbor these organ-/anatomic site-specific commensal and pathogenic microbes (**Figure 2**). Therefore, the intratumoral microbiome should also be organ- or tumor-specific, as it has been observed (97, 194, 195, 227–230).

### 10.3 Integration of microbial DNA into the genome of φTHCs/Mφs

Microbial DNA has been found to be integrated into the host genome (96, 191–193, 200–204). The integration of microbial DNA, particularly viral DNA, can be mediated by transposable elements (202). On the other hand, DNA of microbe-containing apoptotic CaCs is fragmented and relegated following the engulfment by the Mφ/φTHC (Section 4.1 above). The chance of mis-relegating microbial DNA into the rearranged host genome is likely to be high in the presence of defective DNA repair (204, 231), such as in the case of CaCs which are of DNA repair defect (96). Indeed, bacterial DNA is more frequently to be integrated into the genome of CaCs than that of normal cells (200), likely due to DNA repair-dependent genomic instability (232, 233) which can promote the integration of bacterial DNA into the host genome (96, 202, 204).

It should be reminded that only those microbes whose DNA can replicate, either as DNA integrated into the host genome or as extrachromosomal DNA, along with the replication of the host genome, can be detected among most, if not all, of offspring effectors of a dormant memφTHC/metCaC.

## 11 Feedback question: can intratumoral microbes be developed as patient-/tumor-tailored φTHC/memφTHC/metCaC-depleting anti-cancer drugs?

### 11.1 Intratumoral microbes as patient-/tumor-tailored φTHC/memφTHC/metCaC-depleting anti-cancer drugs

Anti-cancer microbial preparations, such as oncolytic viruses and oncolytic bacteria, have been developed to treat cancer. However, they are not organ-/tumor-specific. As a result, a direct injection of anti-cancer microbes into the tumor is often required, which is often hardly or even not approachable to non-superficial tumors under some circumstances, particularly a daily-injection regimen is desired as in the case of Coley’s toxin. However, the organ- and tumor-specific intratumoral microbe and microbiome (Section 10 above) may suggest an alternative way to deliver high-dose microbial preparations to a tumor, which is discussed as follows.

As discussed above (Section 10.2 above), both commensal and pathogenic microbes are often organ- and tumor-specific. Moreover, pathogenic microbes (such as human hepatitis viruses, human papilloma virus, Ebstein-Barr virus, *V. cholerae*, *Salmonella* spp., and *S. pneumoniae*) are often more organotropic and tumoritropic, targeting only specific organs and tumors (235–237). As a result, microbes, particularly live pathogens that can proliferate within the φTHC/Mφ, are often specifically enriched to a high level within an organ populated with that φTHC/Mφ (235, 238). Therefore, organotropic/tumoritropic microbes may enhance the delivery of anti-cancer microbial preparations to a specific organ/tumor, possibly circumventing the requirement of direct injection of microbial preparations into a tumor.

Another approach is to identify microbial genes encoding organotropic/tumoritropic proteins and genetically engineer these genes into the genome of a microbe (such as oncolytic virus and oncolytic bacterium), which may re-direct a microbe from its natural target organ to another un-natural but desired target organ/tumor. Alternatively, HUMAN organ-specific homing molecules, similar those of mouse cells (239), might be genetically engineered into the genome of oncolytic microbes (240).

To summarize, using pathogenic microbes that are identified within a tumor cell (i.e., present intracellularly inside a tumor cell), might potentially enhance the tumoritropism of anti-cancer microbial preparations. It remains to be investigated whether this strategy indeed works.

It should be reminded that the virulence factor of live pathogenic microbes should be inactivated/deleted to reduce the pathogenicity of the microbes before they are administered to cancer patients (235).

A separate issue regarding the efficacy of microbial preparations is that Coley and other physicians at his age showed that not only live bacteria but also DEAD bacteria, even bacterial extracts, induced tumor regression, suggesting that it is the cellular physical constructive constituent (such as MAMPs), rather than the biological or pathological function of live bacteria, induce cancer regression. (The advantage of using live over dead bacteria is that live bacteria proliferate to produce a large number of cellular constituents and hence, a high delivery dosage of bacteria is probably NOT necessary, which could potentially minimize toxicity.) On the other hand, Mφs engulf and process dead and live bacteria not in the same way (241), suggesting that other mechanisms may also be involved.

Additionally, Dr. Coley also found that, unlike their live counterparts, heat-inactivated *Streptococci* lost their anti-cancer activity, which could be restored by adding another heat-inactivated *Serratia* (242). Heat can inactivate a number of molecules. *Serratia* and *Streptococcus* secrete ShlA and pneumolysin, respectively. Both ShlA and pneumolysin are pore-forming toxins and cytolytic to Mφs (243). MOST IMPORTANTLY, these Mφ cytolytic pore-forming toxins could potentially be explored as anti-cancer drugs, either alone or in combination with other microbial preparations such as various types of MAMPs (244). Other pore-forming toxins with relative high specificity towards Mφs and “CaCs” cells, such as MakA from *V. cholerae* (245, 246) and ClyA form *E. coli* and *S. enterica* (247, 248), have also been shown to suppress CaCs (249–251).

Additionally, Mφ-targeting microbes (such as *M. tuberculosis* and *M. Leprae*) or their constituents that pathogenically damage Mφ, may be explored as anti-cancer agents for the φTHC/memφTHC/metCaC. Strategies to enhance their organotropism/CaC specificity are likely demanded.

### 11.2 Selection of types of microbes for patient-/tumor-tailored Mφ-depleting therapy

As discussed above, a tumor harbors many types of microbes, including not only pathogenic but also commensal microbes (97, 193–199). Therefore, it raises a question as to which types of microbes should be selected to specifically deplete the φTHC/memφTHC/metCaC. We speculate that pathogenic microbes (including conditional pathogenic microbes), rather than commensal microbes, should primarily be selected to deplete the φTHC/memφTHC/metCaC. This is because pathogenic microbes are more likely to be the original microbes that elicit the initial inflammation and cause the first-generation Mφ/φTHC. Therefore, comparing to commensal microbes, pathogenic microbes are likely to be more efficacious to deplete the φTHC/memφTHC/metCaC. Commensal microbes that have been found present intracellularly in the φTHC/memφTHC/metCaC are more likely to be engulfed by the φTHC/memφTHC/metCaC as collaterals in a pathogenic microbe-induced inflammation (Section 10.1 above). However, it remains to be verified whether this assumption is true or not, because commensal microbes should theoretically also deplete PRR-specific clones as long as they share the same MAMPs and activate the φTHC/memφTHC/metCaC as the pathogenic microbes do. (This approach requires that the PRR of CaCs should be pre-determined such that microbes with a matched MAMP can be applied.) The advantage of using a commensal microbe is that it is not pathogenic and therefore, is less toxic and much safer. It is remains to be validated whether a microbial preparation combining Mφ-cytolytic pore-forming toxins (Section 11.1 above) from a pathogenic microbe with MAMPs from a commensal microbe, would enhance efficacy and reduce toxicity.

Additional to a microbial preparation composed of a single type of microbe, a cocktail consisting of multiple tumor-specific microbes or microbial constituents (252, 253) may be prepared to treat cancer patients, particularly when their pathogenic microbes cannot be determined.

### 11.3 Hormesis-like effects of microbial and biochemical preparations on tumors

According to the *Memory Macrophage Theory*, the effect of microbes on φTHCs/memφTHCs/metCaCs is dosage-dependent (1). Just like they do to quiescent conventional Mφs/memMφs, lower doses of microbes can awaken dormant/quiescent metCaCs/memφTHCs whereas an excessively high dosage of microbes can deplete φTHCs/metCaCs/memφTHCs (1). This hormesis-like phenomenon of microbes on φTHCs/metCaCs/memφTHCs indicates that a dose-response curve (i.e., a wide range of doses/dosages) of microbes, MAMPs/DAMPs or other PRR-binding ligands is probably necessary to be determined before any “inhibitory” or “stimulatory” effects are concluded, whenever these microbial or biochemical preparations are applied for laboratory research or clinical studies (including clinical trials of anti-cancer/oncolytic microbial preparations).

## 12 Feedback question: can MAMPs and PAMPs be developed as metCaC-depleting preparations?

We have discussed in the original *Memory Macrophage Theory* article (1) that MAMPs and PAMPs can potentially be applied to deplete φTHCs/memφTHCs/memCaCs, which has also been proposed earlies by Hobohm et al (255),. However, a number of issues might be required to be addressed.

A tumor often consists of a number of clones of memφTHCs/memCaCs that express distinct types of PRRs. Each clone expresses a specific type of PRR binding to a specific type of matched MAMP/DAMP (such as receptor TLR9 and its matched ligand double strand DNA) (1, 256). Therefore, a microbe that consists of all MAMPs can presumably deplete all clones of φTHCs/memφTHCs/memCaCs that bear various types of PRRs, whereas a single MAMP is only likely to deplete a limited number of PRR-matching clones of CaCs within a tumor (or a portion of a tumor), without affecting other PRR-unmatching φTHCs/memφTHCs/memCaCs. As a result, it is IMPORTANT that multiple MAMPs/DAMPs (so-called cocktail PRR ligands), which cover all potential PRRs, might be required to deplete all clones of CaCs (254). Under this circumstance, identifying various PRRs of all clones of φTHCs/memφTHCs/memCaCs of a tumor, is probably desired such that matched MAMPs can be applied for immune depletion.

Additional to immune depletion, METRONOMICAL administration of PRR ligands can eradicate mouse cancers (254), which is indictive of immune tolerance that is often initially induced by low doses/dosages of promers) (Section 16 below).

Whether the UNPREDICTABLE anti-cancer effect of lipopolysaccharides (LPSs) and Bacillus Calmette Guerin (BCG) results from their ability to ONLY target limited PRR-specific clones of φTHCs/φTHCs/memCaCs, remains to be clarified.

However, as discussed below (Section 20.1 below), the MAMP/DAMP is not the sole determinant that can bind to PRRs and activate φTHCs/memφTHCs/memCaCs. The specificity of a MAMP/DAMP to activate/deplete a dormant φTHC/memφTHC/metCaC is determined by multiple factors. Therefore, much work seems required when the MAMP/DAMP is applied to deplete φTHCs/memφTHCs/memCaCs.

## 13 Feedback question: can microbes be developed as cancer vaccines and what may be required to be considered for microbial vaccines?

Acute microbial infections have been found to reduce cancer incidence, particularly for those patients who are vaccinated/infected during their early life (254) and for tumors that occur IN THE VICINITY OF the infected site (1, 211). Moreover, inoculation of microbial vaccines, such yellow fever virus, lipopolysaccharides (LPSs), and Bacillus Calmette Guerin (BCG) to persons at their younger ages, can non-specifically prevent the development of a number of types of cancers (260), although these microbes themselves are not necessarily carcinogenic or carcinogenic to a specific cancer-prone organ. On the other hand, acute infections can also drive cancer development (257). The *Memory Macrophage Theory* predicts that whether a parenchymal cell is infected with a MAMP-bearing microbe BEFORE or AFTER it is transformed/cancerized, determines if a conventional memMφ or a memφTHC/memCaC is generated (1). Only the Mφ that has engulfed a transformed/cancerous parenchymal cell, can acquire the mutator phenotype and overgrowth property from the parental transformed/cancerous cell (Section 1 above) (1), and develop into a memφTHC/memCaC. (Normal, un-malignant parenchymal cells do not hold the mutator phenotype and overgrowth property.) Monocytes/Mφs phagocytosing UN-transformed/NON-cancerous parenchymal cells, trans-differentiate into CONVENTIONAL Mφs/memMφs. Moreover, as in the case of adaptive memory lymphocytes, once a conventional Mφ/memMφ primed by a specific MAMP is generated, future exposure to the same MAMP activates this already-existing Mφ/memMφ to cope with any microbes that bear the same specific MAMP, without the necessity/chance for the immune system to REDUNDANTLY generate another brand new Mφ/memMφ (i.e., φTHC/memφTHC/memCaC in this case); redundantly generating another brand new Mφ/memMφ/φTHC/memφTHC/memCaC delays inflammatory/immune responses (1).

Whether this scenario (i.e., vaccination before or after a parenchymal cell is cancerized) attributes to the inconsistent outcome of human papilloma virus vaccines (258, 259) between young and adult females, remains to be determined. Likewise, more studies are required to validate whether this scenario also accounts for previous observations that infections/vaccinations with microbes (such as yellow fever virus, Bacillus Calmette Guerin, and human papillomavirus vaccines) at YOUNGER ages may prevent cancer (260). However, one issue is likely to be true, that is, parenchymal cells of a younger human experience less DNA damage and have a larger chance of being un-transformed/un-cancerous, and hybridization of a monocyte/Mφ with these younger parenchymal cells is more likely to produce a conventional Mφ/memMφ, rather than a φTHC/memφTHC/memCaC. Therefore, vaccinating a person with microbes at younger ages, induces conventional Mφs/memMφs, preventing future generation of φTHCs/memφTHCs/memCaCs.

As a result, several issues related to microbial cancer vaccines are implicated.

### 13.1 Microbes as potential prophylactic vaccines for cancer prevention

As discussed above (this Section above), if inoculated before a parenchymal cell is transformed/cancerized, a vaccine prepared from a tumor-SPECIFIC INTRATUMORAL MICROBE (as determined by family members’ intratumoral microbiome/microbiota and from literature reports for a specific type of tumor) should prevent the development of an organ-specific tumor.

### 13.2 Target patient populations and target patient ages to inoculate microbial vaccines

Individuals who have a family history of a specific type of cancer and/or have a hereditary DNA repair-defective syndrome that is prone to cancer (**Table 2**), are indicated to be inoculated with microbial vaccines of tumor-specific intracellular pathogenic microbes/microbiota, particularly those that have been identified among other family members. Moreover, as discussed above (this Section above), the inoculation should be administered in their early life, BEFORE parenchymal cells are transformed/cancerized.

### 13.3 Vaccines consisting of one strain vs multiple strains of the same type of microbe

Mφs/memMφs/φTHCs/memφTHCs/metCaCs are MAMPs-specific. From the perspective of the specificity of MAMPs ONLY, inoculation of multiple strains of the same species of a microbe is probably NOT necessary, unless additional disparate types of MAMPs are provided by distinct strains. On the other hand, chronic infections persistently release free radicals which can mutate DNA. Moreover, some microbial proteins can interfere with host DNA repair (155–158). Therefore, the necessity of including multiple strains of a microbial species into a vaccine, remains to be further studied and is probably on a case-by-case basis.

It should be reminded that prevention of a microbial infection and prevention of a cancer are separate issues. Distinct strains of a microbe can induce disparate ADAPTIVE immune responses, which are often antigen-/epitope-specific, whereas a φTHC/memφTHC/metCaC is essentially a Mφ that is hybridized with CaCs and is MAMP/DAMP-dependent.

It should also be reminded that administration of a high dosage of a cancer-associated microbial vaccine can potentially THERAPEUTICALLY deplete φTHCs/memφTHCs/metCaCs (Section 16 below) (Sections 11) and treat metastatic diseases (1).

## 14 Feedback question: do conventional Mφs migrate from a peripheral to another non-bone marrow peripheral organ, and do conventional Mφs retro-differentiate of into monocytes?

At the time when we published the *Memory Macrophage Theory* article in July 2024, there seemed no reports in the literature that a monocyte/Mφ can migrate from one peripheral organ to another peripheral organ or to the bone, although memMφs are found in the bone or in non-infected areas of the same peripheral organ (1). Recently, however, it has been reported that MONOCYTES can migrate from the periphery to the bone (261). Additionally, it has also been reported that Mφs can retro-differentiate into MONOCYTES (262). Moreover, MONOCYTES can also acquire memory (263).

Whether the above scenarios (i.e., scenario of retro-differentiation from Mφs to monocytes and scenario of monocytes migrating from a peripheral organ to the bone/another peripheral organ) are solitary cases or general practice for monocytes/Mφs, remains to be studied. It also remains to be investigated whether these scenarios are only limited to memory monocytes/memMφs. In the latter case, the number of these monocytes/Mφs would be limited and difficult to be identified. On the other hand, selective activation of these scarce cells may expand their population, facilitating tracing these cells. The above scenarios also raise a question whether the MONOCYTE is the form for the memMφ to migrate to distant organs and remain in the dormant status. Analysis of chromosome structural variations (99, 100) might answer this question. Recently, it has been found that subsets of monocytes respond distinctly to advanced gastrointestinal cancer and COVID-19 (264).

## 15 Feedback question: can lymphocytes, such as γδT cells, induce apoptosis of conventional Mφs, as well as memφTHCs/metCaC?

As discussed in the *Memory Macrophage Theory* article (1), the relationship between Mφs and lymphocytes is mutually regulatory. Depending on the inflammatory stages (initiation/activation, expansion, or resolution of an inflammation), these two types of immune cells can be mutually cooperative, inhibitory or stimulative (265–267). Furthermore, they can mutually induce apoptosis (268). Indeed, mouse lymphocytes, mediated by FasL, can induce Mφs to undergo apoptosis in the resolution stage of an inflammation (269). Moreover, mouse Vγ1 subtype T lymphocytes were found to contribute to FasL-mediated apoptosis of Mφs (270). As φTHCs/memφTHCs/metCaCs are essentially Mφs that have hybridized with CaCs via phagocytosis or fusion, it is expected that human counterparts of mouse Vγ1 subtype T lymphocytes would also cause apoptosis of φTHCs/memφTHCs/metCaCs.

The relationship between Mφs and lymphocytes seems CELL CLONE-SPECIFIC, mediated by factors such as major histocompatibility complex (MHC) and antibodies (256, 271, 272). Therefore, elucidating the specificity between specific clones of γδT lymphocytes and specific clones of Mφ/memMφ/φTHCs/memφTHCs/metCaCs, might SELECTIVELY kills specific φTHC/memφTHC/metCaC clones without damaging other conventional Mφs, thereby reducing the toxicity ofγδT lymphocyte-dependent immunotherapy. Understanding the specificity between conventional Mφs and lymphocytes may facilitate the development of φTHC/memφTHC/metCaC-specific, γδT lymphocyte-dependent anti-cancer immunotherapy.

It should be reminded again that leukocyte-induced damage of CaCs is often absent or extremely rare (1, 273) Contrarily, tumor-infiltrated lymphocytes may advance tumor progression during the reparative stage of an inflammation (273). This is consistent with earlier observations that M2 tumor-associated macrophage (TAM) are closely related to the generation of the memφTHC/metCaC (125, 126, 131, 132) in the reparative stage of an inflammation (133). Nevertheless, these scenarios once again show the complex relationship between adaptive lymphocytes and innate Mφs/memMφs or innate φTHCs/memφTHCs/metCaCs. Therefore, it is probably not surprising that immune responses can paradoxically either stimulate or inhibit the development of cancer (211).

## 16 Feedback question: should dampening innate immune φTHCs/memφTHCs/metCaCs be considered as a principle to treat cancer patients?

In the *Memory Macrophage Theory* article (1), we primarily discussed that depleting Mφs can treat cancer. We also briefly state that other approaches, such as immune paralysis, anergy, tolerance, exhaustion, etc., which can quantitatively and functionally damage innate immune φTHCs/memφTHCs/metCaCs, can also be explored as anti-cancer strategies to destroy clones of φTHCs/memφTHCs/metCaCs. We speculate that some cases of “spontaneous” regression of tumors following severe microbial infections, could be related to dampening not only the quantity but also the FUNCTION of innate immune φTHCs/memφTHCs/metCaCs. Unlike Coley’s toxin which requires long-term and high-dose/dosage administration of bacterial preparations, severe infections that induce “spontaneous” tumor regression often do not last for months.

Among immune paralysis, anergy, tolerance, exhaustion, etc., immune tolerance has more been investigated. For example, under context-dependent conditions, interferons (IFNs) at APPROPRIATE levels can induce tolerance of monocytes (274). Eradication of solid tumors by METRONOMIC administration of a cocktail composed of PRR ligands/MAMPs, is reminiscent of immune tolerance (254).

PD-1 or PD-L1/PD-L2-targeted therapy can dampen Mφs (15, 16) and hence, φTHCs/memφTHCs/metCaCs (Section 2.4 above) (17). Likewise, T-lymphocyte-mediated programmed cell death of Mφs (Section 15 above) can also quantitatively and functionally dampen φTHCs/memφTHCs/metCaCs. According to the *Memory Macrophage Theory* (1), it is expected that φTHCs/memφTHCs/metCaCs that are more metastatic, should more effectively respond to PD-1 or PD-L1/PD-L2-targeted therapy, because φTHCs/memφTHCs/metCaCs with high metastability are suggestive that these hybrids have acquired more cellular constituents from Mφs/memMφs (Section 8.2).

Additionally, the SPECIFICITY of immune checkpoint inhibitors to SELECTIVELY inhibit/kill φTHC/memφTHC/metCaC clones without interfering with other immune checkpoint molecule-expressing conventional Mφs and immune cells, remains to be improved. As discussed below (Section 20.1 below), elucidating specific interactions between specific clones of innate immune φTHCs/memφTHCs/metCaCs and specific clones of ADAPTIVE immune proteins/cells, may be of value. Moreover, context-dependent immune effects and hormesis-like effects (Section 11.3 above) should be considered.

## 17 Feedback question: what are proper protocols for microbe-based anti-cancer therapy?

We have discussed potential strategies to deliver anti-cancer microbes (such as oncolytic viruses and oncolytic bacteria) specifically to tumors (i.e., organotropism/tumoritropism) (Section 11.1 above). Another issue related to anti-cancer microbes is concerning therapeutic regimens. Currently, therapeutic regimens for oncolytic viruses and oncolytic bacteria are often inherited from those of classic cytotoxic chemotherapy, that is, the interval between two consecutive administrations of microbial preparations is often weeks to months apart. During this long interval, remaining φTHCs/memφTHCs/metCaCs can repopulate and the tumor can grow back (Section 11.1 above). Therefore, to effectively deplete φTHCs/memφTHCs/metCaCs, Dr. Coley’s strategy should probably be adopted, that is, injecting Coley’s toxin to patients every day or every other day for months. Coley’s regimen is indictive of an Mφ-depleting rationale. As a matter of fact, Orange et al. conducted a tremendous amount of work on anti-cancer microbial and related biochemical preparations. Clinicians and biopharmaceutical companies that are pursuing microbial anti-cancer therapy, are STRONGLY recommended to review their work (254).

Dr. Coley stated that fever was critical for the success of Coley’s toxin. Fever suggests the presence of pyrogens which could result from the breakdown of the φTHC/memφTHC/metCaC, additional to microbial endotoxins. Whether fever should be clinically intervened, have been debated (133, 211, 242). According the *Memory Macrophage Theory*, fever is likely to be caused by the release of the endogenous pyrogen of the φTHC/memφTHC/metCaC. Therefore, antipyretic therapy that does NOT interfere with Mφ/memMφ/φTHC/memφTHC/metCaC depletion, is presumably applicable, which may improve the compliance of patients receiving anti-cancer microbial preparations. However, much work is required to confirm this assumption. On the other hand, antibiotics, anti-inflammatory drugs, etc. may delay cancer development (1), suggesting the involvement of microbes and microbe-induced inflammation.

## 18 Feedback question: should Mφ-depleting therapy be applied as an adjunct for surgical excision of primary tumors?

We have proposed in the *Memory Macrophage Theory* article that it might be indicated that the tumor at the primary site be resected because it constantly generates priCaCs and other φTHCs (1). However, the surgical resection of tumors is not necessarily to be conducted BEFORE Mφ-depleting treatment. Like Coley’s toxin (275), pre-surgical depletion of φTHCs might shrink a tumor, facilitating surgical resection of the tumor.

In this regard, whether NON-specific Mφ depleters (276, 277), such as liposomal clodronate and monoclonal antibodies against Mφ, might be used TRANSIENTLY and peri-surgically to alleviate clinical crisis or to facilitate surgery, remains to be investigated although animal studies have shown that NON-specific depletion of Mφ, irrelevant to surgery, can promote metastasis, because some subtypes of Mφs can prevent metastasis (278).

## 19 Feedback question: how can Mφ-depleting therapy be combined with contemporary cytotoxic chemotherapy?

It has been shown that cytotoxic chemotherapy alone promotes the development and metastasis of cancer (279, 280). This presumably results from the breakdown of CaCs, releasing MAMPs/DAMPs that can prime monocytes/Mφs to form φTHCs or awaken dormant/quiescent memφTHCs/metCaCs (1). The broken CaCs can also be phagocytosed by other Mφs/φTHCs, promoting metastasis of φTHCs (1). On the other hand, cytotoxic drugs can also kill CaCs, including φTHCs. The therapeutic outcome, particularly the TEMPORARY outcome, is likely to be determined by compounded effects. It is well-known that dormant/quiescent CaCs (i.e., memφTHCs/metCaCs) are often resistant to cytotoxic therapy and cannot be eradicated by cytotoxic drugs. Therefore, the long-term therapeutic outcome of cytotoxic therapy deserves further evaluation.

It should be reminded again that, to achieve the goal of depleting φTHCs, microbial or biochemical preparations are required to be administered to patients every day or every other day (Section 17 above), which differs from the current chemotherapy regimen; the interval between two consecutive drug-administrating cycles of the current chemotherapy regimen is often several weeks apart. Administration of microbial or biochemical preparations to patients every weeks or months apart, is not likely to deplete φTHCs because residual φTHCs can proliferate to repopulate the tumor during this interval (Section 11.1 above).

## 20 Feedback question: what are unanswered questions?

### 20.1 Contributors to awakening memMφs/memφTHCs/metCaCs

Many MAMPs are often shared by a number of commensal and pathogenic microbes (256). Moreover, humans are exposed to commensal microbes daily and throughout a day. Therefore, if awakening a memφTHC/metCaC is solely determined by MAMPs/DAMPs, the occurrence/recurrence of cancer would be much higher than the actual incidence, suggesting that other factors also contribute to the awakening of the dormant/quiescent memφTHC/metCaC. Indeed, additional to MAMPs/DAMPs, other factors, such as adaptive immunity (256, 271, 272), types of microbes or stimuli (74, 196), live or dead microbes (241), compounded effects of multiple stimuli on gene expression of a Mφ (281), cross-talks among PRRs (282) etc., also regulate specific activities of an innate Mφ/φTHC (196, 281). Identification of these specificity-contributing factors can potentially lead to novel therapeutic strategies and enhance the specificity of Mφ-/φTHC-depleting and other Mφ-/φTHC-dampening therapies.

### 20.2 Self-restrain of Mφs for overgrowth

Like most biological responses, conventional Mφs often turn off proliferation at the end stage of an inflammation (i.e., negative feedback) (74). Therefore, awakened conventional memMφs often do not excessively overgrow within Mφ niches at the metastatic site (such as the bone) (283, 284). However, unlike conventional Mφs/memMφs, φTHCs/memφTHCs/metCaCs overgrow boundlessly not only at the primary organ but also at the metastatic site. This implies that memφTHCs/metCaCs lose their ability to restrain their overgrowth (i.e., negative feedback). The loss of negative feedback on overgrowth of conventional Mφs/memMφs is known to be mediated by a number of mechanisms, including cross-talks between PRRs and inhibitory immunoreceptors (283, 284). Considering that φTHCs/memφTHCs/metCaCs are essentially Mφs/memMφs, whether loss of these mechanisms also leads to the excessive overgrowth of φTHCs/memφTHCs/metCaCs, remains to be verified.

Additionally, unlike conventional Mφs, awakened φTHCs/memφTHCs/metCaCs seem to lose the ability to migrate out of or seem to be prohibited from leaving the metastatic site (i.e., the Mφ/φTHC niche at both primary and distant organs). Migration/metastasis is a chemotactic process involving coordinated efforts of many chemokines and chemokine receptors, as well as interactive immune cells in the microenvironment. Therefore, it may be worth of identifying which one of these participants loses chemotaxis-related functions and whether the loss of the functions is a result of genetic alterations resulting from relegation of apoptotic DNA fragments (Section 4.1 above).

Understanding above phenomena may lead to new anti-cancer strategies.

### 20.3 Clinical phenomena NOT answered by the *Memory Macrophage Theory*

It has been observed that the recurrence of breast cancer shows dual peaks following the resection of the primary tumor (285). The *Memory Macrophage Theory* does NOT seem to have an answer for this phenomenon. Whether this phenomenon is related to the life-span of specific clones of PRR-specific memMφs/memφTHCs/metCaCs, remains to be investigated; the life-span of a Mφ/memMφ and hence, the memφTHC/metCaC is variable, ranging from days to years (1 and references therein). Alternatively, it could also be associated with the so-called immunological dormancy, that is, immune cells (such as memMφs) under dormancy often release sentinels periodically into the blood circulation to patrol for microbial pathogens (1).

## 21 Feedback question: can *Memory Macrophage Theory* also interpret hematological malignancies?

The *Memory Macrophage Theory* primarily addresses the hematogenous metCaC of SOLID tumors. On the other hand, hematological malignancies, such as leukemia, also often occur to individuals with congenital DNA repair-defective syndromes and show mutator phenotypes (**Table 2**) (286–291). Moreover, the development of hematological malignancies commonly involves microbes and integrations of microbial DNA into the host genome (192, 200, 292, 293) as well. Therefore, the possibility of hematological malignancies resulting from the hybridization of a DNA-repair-defective hematological lineage cell with a DNA-repair-defective Mφ or an innate lymphoid cell (ILC) that possesses phagocytosing ability, cannot be excluded. Indeed, fusion between Mφs and other hematological lineage cells is suspected to contribute to hematological malignancies such as multiple myeloma (294) and Hodgkin’s disease (295). Additionally, *in vivo* animal studies have shown that Mφs confer the metastatic ability to non-metastatic hematological tumor cells (239, 296). Therefore, it again suggests the importance of hybridization between a transformed/cancerized cell and a Mφ, as well as the importance of erroneous processing of apoptotic DNA of the hybrid cell by defective DNA repair (Section 4.1), to the development of malignancies.

## 22 Feedback question: what should be considered to study φTHCs/memφTHCs/metCaCs for bench scientists and clinicians?

### 22.1 Necessity of labeling with dual biomarkers of Mφs/memMφs and priCaCs for studying CaCs/φTHCs/metCaCs/memφTHCs as well as tumor-associated macrophages

As discussed above (Section 5 above), up to ~100% of circulating tumor cells (CTCs), disseminated tumor cells (DTCs), and/or metCaCs at the primary and metastatic sites, are positive for Mφ biomarkers (5, 132, 178), which is consistent with the *Memory Macrophage Theory*. Mφ biomarker-negative CaCs should be found within a primary tumor because the primary tumor is primarily composed of tissue-resident φTHCs/metCaCs/memφTHCs, additional to priCaCs (1).

A tumor consists of various types/subtypes of Mφs, including TAMs, φTHCs/memφTHCs/metCaCs (which are essentially Mφs that have hybridized with CaCs via phagocytosis or fusion), M1-type Mφs, various subtypes of M2 Mφs, etc., as well as ontogenically distinct Mφs (i.e., embryo-derived local Mφs and recruited central/bone marrow monocytes/Mφs) (297, 298). Therefore, labeling a tumor with Mφ biomarkers only, but without biomarkers for priCaCs and φTHCs/memφTHC/metCaC hybrids, is likely to count φTHCs/memφTHCs/metCaCs as “TAMs” (5, 178, 299, 300). *Vice versa*, analyzing a tumor only with priCaC biomarkers can neglect contributions of Mφs to φTHCs/memφTHCs/metCaCs. Therefore, it is probably necessary to dual-label every so-called “TAM” or “CaC” with dual biomarkers (such as surface molecules, RNA, and DNA) of both Mφs and priCaCs. It is also probably necessary to experimentally separate true TAMs from φTHCs/memφTHCs/metCaCs to conduct any biochemical and biological analyses on φTHCs/memφTHCs/metCaCs before attributing any observed phenomena solely to “TAMs” or solely to “CaCs” (299, 300). Indeed, studies on φTHCs/memφTHCs/metCaCs with biomarkers for Mφs/memMφs have found that most, if not all, φTHCs/memφTHCs/metCaCs express Mφ surface molecules (4, 101, 177, 181, 299). Single-cell analysis should also address this issue.

As a matter of fact, φTHCs/memφTHCs/metCaCs are proposed to be originated from the fusion of CaCs with tumor associated macrophages (TAMs) (125, 131).

Additionally, different φTHCs/memφTHCs/metCaCs may express distinct Mφ biomarkers. That is, a φTHC/memφTHC/metCaC may not express ALL Mφ biomarkers for the following reasons. [I] As discussed in the *Memory Macrophage Theory* article (1), as a hybrid, the memφTHC/metCaC consists of both Mφ and priCaC cellular constituents. The proportion that the parental Mφ and priCaC contribute to every memφTHC/metCaC, varies from one memφTHC/metCaC to another. [II] Multi-step carcinogenesis and field cancerization suggest that memφTHCs/metCaCs at later steps of carcinogenesis often consist of more Mφ constituents (Section 8.2 above) and that different CaCs within the same tumor may express distinct Mφ biomarkers. [III] At different stages of a cancer-related inflammation, Mφs/φTHCs can phenotypically behave as M1-type Mφs and/or M2-type Mφs, both of which bear distinct type-specific biomarkers (301, 302). [IV] Different types of cancers may express different Mφ biomarkers (177). To conclude, not all Mφ biomarkers are likely to be expressed by any single φTHC/memφTHC/metCaC. Labeling memφTHCs/metCaCs with MULTIPLE Mφ biomarkers, even general leukocyte biomarkers, such as CD45, might be required for the identification and analysis of all φTHCs/memφTHCs/metCaCs.

### 22.2 Specimens selected for studying CaCs/φTHCs/metCaCs/memφTHCs

Mφs are known to be extraordinarily plastic. Their phenotypes can change along with microenvironmental cues. Additional to M1 Mφs, various subtypes of M2 Mφs, and TAMs, Mφs have also been found to trans-differentiate into epithelioid cells or fibroblasts (211–214) (Section 8.2 above). Likewise, metCaCs/memφTHCs share these properties with Mφs (Section 8.2 above) (**Table 1**) (217). Therefore, *in vitro* medium-cultured cells that have been passaged for generations, could lose the property acquired from parental Mφs (111, 125, 148). As a matter of fact, *L. monocytogene* can induce trans-differentiation of malignant melanocytes into professional antigen-presentation cells (APCs), which mediates regression and eradication of tumors (303). As a result, whenever possible, patient samples, primary cultured cells, or medium-cultured cells that have not been passaged for generations, should probably be selected to study φTHCs/metCaCs/memφTHCs. Whether *in vitro* medium-cultured cells that have been passaged for many generations may lose macrophagic phenotypes morphologically, biochemically, and biologically, or whether these aged cells are of choice, remains to be investigated.

### 22.3 Necessity of defining dose-response curve for microbial and PRR-targeting anti-cancer drugs for clinical and laboratory investigations on metCaCs/memφTHCs

As discussed above (Section 11.3), there is a hormesis-like effect of microbes or their constituents (such as MAPMs) on metCaCs/memφTHCs. Therefore, a dose-response curve is probably necessary to be conducted for any microbial or biochemical preparations tested for anti-cancer effects for laboratory animal studies and clinical trials before these preparations are concluded to be of pro-cancer or anti-cancer.

### 22.4 Allogeneically transplanted organs and fluorescent proteins for studying metCaCs/memφTHCs

As both parental CaCs and Mφs belong to the same patient and share the same genetic background, it is often difficult to differentiate that a DNA segment found in the hybridized φTHC/metCaC/memφTHC (i.e., homotypic hybrid cell) is inherited from the parental priCaC or from the parental Mφ. Clinically, tumors developed within an allogeneically transplanted organ can sometimes resolve such difficulties (106, 127, 148, 179, 304, 305).

Techniques of fluorescent proteins are potentially valuable for laboratory animal and cellular studies to identify source parental cells for DNA that contributes to the genome of the φTHC/metCaC/memφTHC, as well as to trace/track the migratory/metastatic route of the metCaC/memφTHC (306). Distinct parental cells can be labeled with different fluorescent proteins (such as red vs. green ones). However, heterologous DNA encoding fluorescent proteins, as well as the encoded fluorescent proteins, is subjected to degradation following the phagocytosis by the parental effector Mφ. To avoid such possibilities, both host genomic site at which the heterologous DNA is introduced and the copy number (i.e., more than one copies at several host genomic sites) of the heterologous DNA should be considered.

Alternatively, mutated/rearranged DNA of the metCaC/memφTHC (1, 99, 100), as well as the intratumoral microbial DNA integrated into the genome of the metCaC/memφTHC (Section 10.3 above), can be used as surrogate biomarkers to track the metCaC/memφTHC. In this case, no additional genetic engineering is required, comparing to the mothed using fluorescent proteins.

### 22.5 Animal model with spontaneous vs. transplanted metastatic tumors for studying metCaCs/memφTHCs

The *Memory Macrophage Theory* states that the metCaC/memφTHC results from the hybridization between an autologous priCaC and an autologous Mφ (1). The φTHC/metCaC/memφTHC essentially belongs to innate immune cells. Therefore, immune responses of patients or animals to SPONTANEOUS tumors and SPONTANEOUS metastatic tumors, are likely to differ from those animals, even nude mice, that are TRANSPLANTED with allogenous and heterologous tumors. Transplanted tumors can elicit ADAPTIVE immune responses other than INNATE immunity. As a result, translating data from studies on animal models with TRANSPLANTED metastatic tumors, rather than SPONTANEOUS tumors, should probably consider these additional immune responses. Indeed, metCaCs of Mφ origins are generally not found in rodent tumor TRANSPLANT models (4).

On the other hand, as described below (Section 23), zebrafish larvae might be an important model for studying relationships among immune responses, cancer, and microbial infections (234, 307).

## 23 Feedback question: how can *Memory Macrophage Theory* be validated or falsified?

An *in vivo* live imaging study that can directly track/trace the entire metastatic process/cascade of the CaC-Mφ hybrid (i.e., metCaC/memφTHC), would be a more persuasive or less disputable piece of evidence to validate/falsify the *Memory Macrophage Theory*, which is discussed as follows.

### 23.1 Zebrafish larvae as a model to track/trace metCaCs/memφTHCs with *in vivo* live imaging

*In vivo* imaging to study cancer and its metastasis has been developed for a number of animal models, including mice and zebrafishes/zebrafish larvae (234, 307–309). Here, we only discuss zebrafish/zebrafish larva models for their feasibilities (**Box 3**) although mouse models more closely resemble humans.

Box 3 **Advantages of zebrafish/zebrafish larva models for studies on metastasis**

Zebrafishes/zebrafish larvae are of optimal transparency that facilitates high-resolution and real-time *in vivo* live imaging, genetic tractability/traceability, conserved innate and adaptive immune systems between zebrafishes and humans with immaturely developed adaptive immunity at early development, MAMP-/DAMP-activatable PRRs (such as TLRs) expressed by Mφs, availability of spontaneous transgenic tumor models, a so-called conditional cancer toolbox that enables controlled expression of oncogenes and silencing of tumor suppressor genes, availability of fluorescent reporter zebrafishes/zebrafish larvae that express GFP or RFP under the control of Mφ (mpag1) and other cells, viral and bacterial infectability/injection, a chemically induced inflammation (ChIn) assay that can induce and evaluate acute inflammation (307). Therefore, the zebrafish/zebrafish larva model has been applied to investigate metastasis (309) and immune-oncology, such as CAR-T-cell therapy and immune checkpoint inhibitors (307). Additionally, they are cost-effective (307).

Using zebrafish/zebrafish larva models, it has been found that transferring of cytoplasm from a subset of Mφs to CaCs following a prolonged contact between these two types of cells, promotes dissemination of the CaCs (308). Zebrafish/zebrafish larva models have also been used to validate Coley’s toxin- and *E. coli*-induced regressions of pre-neoplasia (234).

Two lines of SYNGENEIC zebrafishes/zebrafish larvae are suggested for *Theory*-validating/falsifying experiments: a CaC donor and a CaC recipient. The SYNGENEIC background for both lines is important as discussed above (Section 22.5 above).

A spontaneous cancer can be induced with an oncogene and/or tumor suppressor gene-transgenic line, as reported in the literature (**Box 3**) (307, 309). The CaC should be fluorescently labeled with GFP or RFP for the convenience of tracking/tracing. The label should be different from the label of the recipient Mφ to enable monitoring hybridization between the CaC and the Mφ. The recipient Mφs can be a line such as mpeg1:mCherry-tagged Mφs (307) whose tag facilitates tracking/tracing as well.

The SYNGENEIC CaC from the donor is planted into the recipient. Hybridization (either fusion or phagocytosis) and metastasis/migration can be studied as previously reported (309). Results of these experiments should validate or falsify the *Memory Macrophage Theory*.

## 24 Summary

**Table 3** summarizes our responses to other feedback.

**Table 3:** Testable predictions that can either validate or falsify the *Memory Macrophage Theory* for cancer metastasis.

Claim	Prediction	Key experiment	Supportive result	Falsifier/question	Comment/answer
The hematogenous metCaC is a hybrid cell between a CaC and memMφ (i.e., memφTHC).	MetCaCs should express Mφ surface biomarkers.	Analyzing metCaCs with Mφ surface biomarkers (Sections 5 and 22.1).	Up to ~100% of cancer cells at primary sites, circulating within blood, and/or at metastatic sites express Mφ surface biomarkers (Sections 5 and 22.1).	Why do not all φTHCs/memφTHCs/metCaCs of a tumor express Mφ surface biomarkers?	[I] Mφ surface biomarker-positive metCaCs can be under-estimated under some experiment conditions (such as analyzing CaCs with only 1 or 2 Mφs surface biomarkers, which cannot identify various subtypes of Mφs and other highly plastic Mφs) (Section 22.1). [II] THCs hybridized with other non-Mφ types of cells (such as MSCs and fibroblasts) can also contribute to metastasis (Section 24). [III] Lymphogenous, not hematogenous, metCaCs may not be memφTHCs even though Mφs are involved (1). [IV] A tumor primarily consists of two types of cancer cells, priCaCs and φTHCs/memφTHCs/metCaCs (1). priCaCs do not supposedly express Mφ surface biomarkers, nor are any φTHCs (1).
	A metCaC/memφTHC should behave like a Mφ/memMφbiochemically, biochemically, and immunologically.	Comparing biochemical, biochemical, and immunological phenotypes between metCaCs and Mφs/memMφs.	**True (Table 1)**.		Mutator phenotypes and inability to leave storage/metastatic sites of φTHCs/memφTHCs/metCaCs are acquired from priCaCs, which are found for conventional Mφs/memMφs (Section 20).
	Fusing immobile CaCs with Mφs should lead to metastasis of these previously immobile CaCs.	Fusing/hybridizing immobile CaCs with Mφs, and analyzing metastability of CaC-Mφ fusion/hybrid cells.	True (1 and references therein, 106, 120, 239, 296).		
Essentially as innate immune cells, THCs/memφTHCs/metCaCs can phagocytose microbes.	Microbial constituents should be found inside metCaCs and microbial DNA could be integrated into metCaCs’ genome, just like they are for Mφs/memMφs.	Analyzing microbes and microbial DNA within φmetCaCs.	True (Sections 6.2 and 10.3).		
Essentially as innate immune cells, dormant memφTHCs/metCaCs can be awakened by an acquainted microbe or MAMP/DAMP primer.	A MAMP-mateched microbial infection should awaken dormant metCaCs, causing recurrence/” occurrence” of metastatic diseases.	Investigating whether microbial infection can awaken dormant/quiescent metCaCs.	True (1 and reference therein).	If an acquainted microbe or MAMP/DAMP primer can awaken dormant memφTHCs/metCaCs, why tumor recurrence/”occurrence” is not often as microbial infections?	Although required for awakening dormant memφTHCs/metCaCs, an acquainted microbe or MAMP/DAMP primer ALONE is NOT sufficient. Other factors, such as a proper dosage, matched PRRs, associated adaptive immune responses (Section 20.1), are necessary.
Essentially as innate immune cells, memφTHCs/metCaCs can also inversely be depleted by MAMPs/DAMPs.	Tumor-/CaC-specific intracellular microbes or other microbes bearing the same MAMPs, should depleteφTHCs/memφTHCs/metCaCs at excessively high dosages, just like they do to conventional Mφs/memφs. Depleting therapy can lead to tumor regression.	Investigating whether high dosages of microbial preparations can kill or eradiate CaCs.	High dosages of microbial preparations, such as Coley’s toxin, kill or eradiate CaCs (1 and references therein).	Why anticancer activities of contemporary microbe-based preparations are marginal and cannot reproduce Coley’s results, including cure for late-stage patients?	[I] Coley injected his toxin daily or every other day for months, followed by weekly injection for additional months, which can deplete φTHCs/memφTHCs/metCaCs. However, contemporary oncolytic viruses/bacteria under clinical trials are often administered once every weeks to be aligned with combination chemotherapy with cytotoxic drugs and/or other immunotherapy. During this lag, φTHCs/memφTHCs/metCaCs proliferate to re-populate tumors. [II] Direct injection can deliver a high dose of microbes into a tumor whereas levels of microbes inside a tumor is minimal when systemic administration, such routine intravenous injection, is administered. [III] Currently, anticancer activities of microbial preparations are considered to be via “boosting immunity” without any SPECIFIC involved “immunity” being definitively validated. However, it is known that anticancer activities of microbes are mediated by activating PRRs. PRRs are specific receptors of Mφs. [IV] “Boosting immunity”, such as vaccination, often does NOT require daily injection of microbes into patients for months. Coley’s regimen does.
	Organ-/tumor-specific intratumoral microbe inside a CaC, should boost efficacy of microbe-based anticancer preparations.	Identifying intracellular microbes inside CaCs from a specific tumor, and treating their anticancer activities *in vivo* with excessive dosages of these microbes.	A tumor regresses although a hormesis-like effect could occur if initial dosages are not sufficiently high (Section 11.3).	A falsifier would be that a tumor never regresses, which is neither true for Coley’s toxin nor for oncolytic microbes.	
Microbial vaccines can prevent an UN-transformed cell from cancerization whereas promote carcer development of a transformed cell.	Vaccination with common tumor-specific intratumoral microbes identified within tumor cells, can prevent tumors of a specific organ.	Vaccinating with organ-/tumor specific microbes to individuals without transformed/cancerized parenchymal cells, and monitoring cancer-preventive effects of organ-specific and microbe-specific cancer.	True (Section 13).		Contrary to the young, the vaccinated non-young is presumably to be more likely to develop than to prevent cancer (Section 13).

Importantly, the current *Memory Macrophage Theory* seems to have answers to many biological and clinical questions, particularly on metCaCs that metastasize to the brain, liver, lungs, and bone which are highly populated with morphologically unique macrophages such as microglia, Kupffer cells, alveolar macrophages, and osteomac/osteoclasts, respectively. On the other hand, cancer is a complex disease. There are not sufficient data that the current *Memory Macrophage Theory* can answer: “Why surgical resection of the primary tumor can sometime lead to regression of the distant metastatic disease? (1)” and “Why breast cancer patients present a dual-peak recurrence following surgical resection of the primary disease? (Section 20.3 above)”. Moreover, metastasis of CaCs to the lymph node is probably not related to memφTHCs (1) although Mφs are involved. Additionally, CaCs can fuse with other types of cells such as mesenchymal stem cells (MSCs) and fibroblasts (101). These fusion cells can also promote metastasis (310). Therefore, the *Memory Macrophage Theory* is only proposed to address the hematogenous metastasis of some CaCs that are hybridized with Mφs and metastasize to organs that are highly populated with various types of Mφs. Whether the *Theory* is true or not, remains to be further validated.
